# PNPLA3 GG Genotype and Carotid Atherosclerosis in Patients with Non-Alcoholic Fatty Liver Disease

**DOI:** 10.1371/journal.pone.0074089

**Published:** 2013-09-17

**Authors:** Salvatore Petta, Luca Valenti, Giulio Marchesini, Vito Di Marco, Anna Licata, Calogero Cammà, Maria Rosa Barcellona, Daniela Cabibi, Benedetta Donati, Anna Fracanzani, Stefania Grimaudo, Gaspare Parrinello, Rosaria Maria Pipitone, Daniele Torres, Silvia Fargion, Giuseppe Licata, Antonio Craxì

**Affiliations:** 1 Di.Bi.M.I.S, Sezione di Gastroenterologia, Università di Palermo, Palermo, Italia; 2 Department of Pathophysiology and Transplantation, Section Internal Medicine, Università degli Studi, Fondazione Ca’ Granda IRCCS Ospedale Maggiore Policlinico, Milano, Italy; 3 Dipartimento Biomedico di Medicina Interna e Specialistica (Di.Bi.M.I.S), Università di Palermo, Palermo, Italia; 4 Dipartimento di Patologia umana, Università di Palermo, Palermo, Italia; 5 Dipartimento di Medicina e Gastroenterologia, “Alma Mater Studiorum,” Università di Bologna, Bologna, Italy; University of Modena & Reggio Emilia, Italy

## Abstract

**Background and Aim:**

To evaluate if the presence of carotid atherosclerosis in patients with NAFLD, could be related to gene variants influencing hepatic fat accumulation and the severity of liver damage.

**Methods:**

We recorded anthropometric, metabolic and histological data(Kleiner score) of 162 consecutive, biopsy-proven Sicilian NAFLD patients. Intima-media thickness(IMT), IMT thickening(IMT≥1 mm) and carotid plaques(focal thickening of >1.3 mm at the level of common carotid artery) were evaluated using ultrasonography. IL28B rs12979860 C>T, PNPLA3 rs738409 C>G, GCKR rs780094 C>T, LYPLAL1 rs12137855 C>T, and NCAN rs2228603 C>T single nucleotide polymorphisms were also assessed. The results were validated in a cohort of 267 subjects with clinical or histological diagnosis of NAFLD from Northern Italy, 63 of whom had follow-up examinations.

**Results:**

Carotid plaques, IMT thickening and mean maximum IMT were similar in the two cohorts, whereas the prevalence of diabetes, obesity, NASH, and PNPLA3 GG polymorphism(21%*vs.*13%, p = 0.02) were significantly higher in the Sicilian cohort. In this cohort, the prevalence of carotid plaques and IMT thickening was higher in PNPLA3 GG compared to CC/CG genotype(53%*vs.*32%, p = 0.02; 62%*vs.*28%, p<0.001, respectively). These associations were confirmed at multivariate analyses (OR2.94;95%C.I. 1.12–7.71, p = 0.02, and OR4.11;95%C.I. 1.69–9.96, p = 0.002, respectively), although have been observed only in patients <50years. Also in the validation cohort, PNPLA3 GG genotype was independently associated with IMT thickening in younger patients only (OR: 6.00,95%C.I. 1.36–29, p = 0.01), and to IMT progression (p = 0.05) in patients with follow-up examinations.

**Conclusion:**

PNPLA3 GG genotype is associated with higher severity of carotid atherosclerosis in younger patients with NAFLD. Mechanisms underlying this association, and its clinical relevance need further investigations.

## Introduction

Nonalcoholic fatty liver disease (NAFLD) affects about 20%–30% of the general population. [1] In addition to being at risk for non-alcoholic steatohepatitis (NASH), leading to cirrhosis and its complications, [1] NAFLD patients are also at higher risk of systemic and cardiovascular diseases. [2] Specifically, surrogate markers of NAFLD, namely fatty liver index, [3] and elevated ALT [4] and GGT [5] have been associated with carotid atherosclerosis and incident cardiovascular disease, respectively. Similarly, NAFLD, diagnosed either by ultrasonography or by liver biopsy, has been associated with a higher prevalence of early asymptomatic cardiovascular alterations such as altered left ventricular geometry and early features of left ventricular diastolic dysfunction, [6], [7] low coronary flow reserve [8] and coronary calcification, [9] and carotid atherosclerosis.[10]–[12] These alterations have been partly associated with the severity of liver damage, measured by both lobular inflammation and fibrosis. Cross sectional studies demonstrated a link between NAFLD and the presence/extent of coronary, cerebral and peripheral cardiovascular involvement, [13] whereas prospective studies identified NAFLD as risk factor for cardiovascular events after correction for conventional confounders. [14].

In the last few years, data arising from genome-wide (GWAS) or candidate association studies identified single nucleotide polymorphisms (SNPs) in genes involved in metabolic homeostasis, inflammation, oxidative stress and fibrogenesis, as associated with NAFLD and its severity. Patatin-like phospholipase-3 (PNPLA3)/adiponutrin, rs738409 C>G SNP, remains the most validated risk gene in this setting. [15], [16] Because NAFLD is the liver expression of a systemic metabolic dysregulation, we hypothesized that SNPs of genes associated with NAFLD and its severity -PNPLA3, GWAS-derived genes like neurocan (NCAN), glucokinase regulatory protein (GCKR), lysophospholipase-like 1 (LYPLAL1) [17], and IL28B which have been recently associated with hepatic lobular inflammation and fibrosis [18] - could also be linked to the severity of systemic, and in particular cardiovascular alterations in this setting of patients.

We evaluated carotid atherosclerosis in an homogeneous cohort of biopsy-proven NAFLD patients, in order to assess its correlation with gene variants influencing hepatic fat accumulation and the severity of liver damage in NAFLD. The results were raised and validated in two totally independent cohorts.

## Materials and Methods

The study was performed in accordance with the principles of the Declaration of Helsinki and its appendices, and with local and national laws. Approval was obtained from the AOUP Policlinico Paolo Giaccone of Palermo and from the Fondazione IRCCS Ca’ Granda Ospedale Maggiore Policlinico Milan, Institutional Review Boards and Ethics Committees, and written informed consent was obtained from all patients.

### Patients

We analyzed data from 429 Italian consecutive patients with NAFLD and with available DNA and ultrasonographic carotid arteries assessment. Among them, 162 patients with clinical and histological diagnosis of NAFLD were prospectively recruited at the Gastrointestinal & Liver Unit of the Palermo University Hospital (training set)., and 267 patients were recruited at the Metabolic Liver Diseases outpatient service, Fondazione IRCCS Ca’ Granda Ospedale Maggiore Policlinico, Milan (validation set),. The latter has already been included in a previous study on cardiovascular risk factors for NAFLD. [19] Other causes of liver disease were excluded, including alcohol intake (>20 g/day) evaluated by a questionnaire, viral and autoimmune hepatitis, hereditary hemochromatosis, and alpha1-antitrypsin deficiency. Patients with advanced cirrhosis, hepatocellular carcinoma, current use of drugs interfering with lipid metabolism (i.e., statins, fibrates) (only for Sicilian patients), and previous diagnosis of carotid atherosclerosis, were excluded.

### Clinical, Laboratory and Histological Assessment

Clinical and anthropometric data, including BMI, the presence of arterial hypertension and type 2 diabetes, were collected at the time of enrollment. [20] A 12-hour overnight fasting blood sample was drawn to determine serum levels of ALT, total, HDL and LDL-cholesterol, triglycerides, ferritin, GGT, plasma glucose concentration, and insulin. Insulin resistance was determined by the homeostasis model assessment (HOMA). [21] Serum adiponectin (SPIbio – Bertin Pharma Human Adiponectin EIA Kit) levels were measured in duplicate in the Sicilian patients.

Slides were coded and read at each clinical center by one expert pathologist, who was unaware of patients identity and history. A minimum 15 mm-length of the biopsy specimen or the presence of at least 10 complete portal tracts was required. [22] Steatosis was assessed as the percentage of hepatocytes containing fat droplets (minimum 5%), and evaluated as a continuous variable. Kleiner classification [23] was used to compute the NAFLD activity score (NAS) (from 0 to 8, on a scale including separate scores for steatosis, lobular inflammation, and hepatocellular ballooning) and to stage fibrosis from 0 to 4. NASH was considered to be present when steatosis, ballooning, and lobular inflammation were present.

### Genetic Analyses

DNA was extracted from peripheral blood collected at the time of enrollment in both Sicilian and Northern Italy cohorts.

Genotyping for IL28B (rs12979860), PNPLA3 (rs738409), GCKR (rs780094), LYPLAL1 (rs12137855), and NCAN (rs2228603) was carried out using the TaqMan SNP genotyping allelic discrimination method (Applied Biosystems, Foster City, CA, USA). Commercial genotyping assays were available for the following SNPs: rs738409 (cat. C_7241_10), rs780094 (cat. C_2862873_10), rs12137855 (cat. C_31403184_10), rs2228603 (cat. C_16171492_10). A custom assay was created by Applied Biosystem for rs12979860. The genotyping call was done with SDS software v.1.3.0 (ABI Prism 7500, Foster City, CA, USA).

Genotyping was conducted in a blinded fashion relative to patient characteristics.

### Carotid Artery Evaluation

Carotid atherosclerosis was evaluated by an expert physician in each clinical centre, in a blinded fashion as to characteristic of patients, using a high-resolution B-mode ultrasonography equipped with a multifrequency linear probe.

Carotid arteries were investigated in longitudinal projections of both the left and right side at the level of the common carotid artery, of the bulb and of the internal carotid. The carotid intima-media thickness (IMT) was measured as the difference between the first (intima-lumen) and the second (media-adventitia) interface on the far wall of the common carotid artery in a section free of plaques beginning 10 mm below their bifurcations and including the bifurcations for 10 mm. For each subject, three measurements on both sides were performed, i.e., the anterior, lateral, and posterior projection of the near and far wall. Maximum (outside the plaque) rather than mean values of IMT were considered, and edge detection was performed manually. IMT measurements from the left and right side were averaged. Carotid thickening was defined as an IMT≥1 mm; this value has been previously associated with a higher cardiovascular risk. [24], [25] A carotid plaque was defined as a focal thickening >1.3 mm at the level of either common and internal carotid arteries or bifurcations. [12].

### Statistics

Continuous variables were summarized as mean ± SD, and categorical variables as frequency and percentage. The ANOVA test, student’s t-test and chi-square test were used when appropriate.

Multiple logistic regression models were used to assess the relationship of IMT thickening (≥1 mm), and carotid plaques with clinical, biochemical, genetic (PNPLA3 only in Milan patients) and histological parameters in both NAFLD cohorts. In the first model, the dependent variable was the presence of an IMT thickening coded as 0 = IMT<1 mm vs 1 = IMT≥1. In the second model, the dependent variable was the presence of one or more carotid plaque, coded as 1 = present vs. 0 = absent. As candidate risk factors, we selected age, gender, BMI, baseline ALT, triglycerides, total, HDL and LDL cholesterol, GGT, ferritin, blood glucose, diabetes, arterial hypertension, smoking, insulin, HOMA, adiponectin (only in Palermo patients), IL28B rs12979860 (only in Sicilian patients), PNPLA3 rs738409, GCKR rs780094 (only in Sicilian patients), LYPLAL1 rs12137855 (only in Sicilian patients), NCAN rs2228603 (only in Sicilian patients), and histological variables like steatosis, lobular inflammation, hepatocellular ballooning, NAS score, NASH, and fibrosis (for all Sicilian patients, and for the Northern Italian patients who underwent liver biopsy).

Multiple linear regression analysis was done to identify independent predictors of IMT (continuous dependent variable) in both cohorts of NAFLD patients. As candidate risk factors, we selected the same independent variables included in the logistic model.

In all models, we compared patients homozygous for at-risk alleles to all other variants. [15].

Regression analyses were done using Proc Logistic, Proc Reg, and subroutine in SAS (SAS Institute, Inc., Cary, North Carolina, U.S.A.). [26].

## Results

### Patient Features and Histology

The baseline features of the 162 Sicilian (training set) and the 267 Northern Italian (validation set) patients are shown in Table 1. Sicilian patients were younger, comprised a larger prevalence of females and of subjects with metabolic alterations (obesity and diabetes), and had higher ALT serum levels compared to Northern Italian patients.

**Table 1:** Baseline Demographic, Laboratory, Metabolic, Genetic and Histological Features of 429 Italian Patients with Non-alcoholic Fatty Liver Disease.

Variable	Non-alcoholic Fatty LiverDisease (Sicily n = 162)	Non-alcoholic Fatty LiverDisease (Northern Italy n = 267)	P value
**Mean Age** – **years**	47.2±12.8	52.9±12.4	<0.001
**Age≥50 years**	74 (45.7)	154 (57.6)	0.02
**Male Gender**	102 (63)	217 (81)	<0.001
**Mean Body Mass Index** – **Kg/m^2^**	30.0±4.9	27.5±7.9	<0.001
**Body Mass Index** – **Kg/m^2^**			
<25	20 (12.3)	81 (30.3)	
25–29.9	70 (43.2)	137 (51.3)	
≥30	72 (44.5)	49 (18.4)	<0.001
**Arterial Hypertension**	46 (28.4)	93 (34.8)	0.20
**Type 2 Diabetes**	29 (17.9)	21 (8.0)	0.002
**Smoking**	27 (16.7)	72 (27.0)	0.01
**Statin use**	0 (0)	22 (8)	<0.001
**Carotid Intima-Media Thickness – mm**	0.85±0.24	0.86±0.23	0.66
**Carotid thickening**	57 (35.2)	90 (33.7)	0.83
**Carotid Plaques**	59 (36.4)	84 (31)	0.29
**Alanine Aminotransferase – IU/mL**	77±48	39±25	<0.001
**Cholesterol – mg/Dl**	205.6±47.0	202.6±33.7	0.44
**HDL Cholesterol – mg/Dl**	51±19	43±14	<0.001
**LDL Cholesterol – mg/Dl**	126±39	126±33	0.99
**Triglycerides – mg/Dl**	145±77	139±74	0.37
**Blood Glucose – mg/Dl**	99.0±28.2	95.2±16.3	0.07
**Insulin – µU/Ml**	16.5±9.7	16.9±10.3	0.69
**HOMA Score**	4.14±3.14	4.2±3.7	0.86
**Hyperferritinemia**	52 (32.1)	121 (45.3)	0.009
**IL28B rs12979860 polymorphism**			
C/C	74 (45.7)	–	
T/C	74 (45.7)	–	
T/T	14 (8.6)	–	–
**PNPLA3 rs738409 polymorphism**			
C/C	53 (32.7)	113 (42)	
C/G	75 (46.3)	121 (45)	
G/G	34 (21.0)	33 (13)	0.02
**GCKR rs780094** **polymorphism**			
C/C	24 (14.8)	–	
C/T	84 (51.9)	–	
T/T	54 (33.3)	–	–
**LYPLAL1 rs12137855 polymorphism**			
C/C	95 (58.6)	–	
C/T	64 (39.5)	–	
T/T	3 (1.9)	–	–
**NCAN rs2228603** **polymorphism**			
C/C	145 (89.5)	–	
C/T	17 (10.5)	–	
T/T	0 (0.0)	–	–
**Histology** *			
** NAFLD activity score (NAS)**			
1–2	17 (10.5)	97 (76.0)	
3–4	44 (27.2)	23 (18.3)	
5–8	101 (62.3)	7 (5.6)	<0.001
** Lobular inflammation**			
0	12 (7.4)	50 (39.7)	
1	77 (47.5)	70 (55.6)	
2	66 (40.7)	6 (2.7)	
3	7 (4.3)	0	<0.001
** Steatosis grade**			
1 (5%–33%)	57 (35.2)	74 (58.7)	
2 (>33%–66%)	49 (30.2)	40 (31.7)	
3 (>66%)	56 (34.6)	12 (9.6)	<0.001
** Hepatocellular ballooning**			
0	13 (8.0)	103 (87.7)	
1	75 (46.3)	23 (22.3)	
2	74 (45.7)	0	<0.001
** NASH**			
present	160 (98.8)	58 (46)	
absent	2 (1.2)	68 (54)	<0.001
** Stage of Fibrosis**			
** 0**	45 (27.8)	58 (46.0)	
** 1**	39 (24.1)	43 (34.2)	
** 2**	38 (23.5)	18 (14.2)	
** 3**	27 (16.7)	3 (2.3)	
** 4**	13 (8.0)	4 (3.2)	<0.001

Abbreviation: IU, international units; HOMA, homeostasis model assessment; LDL, low density lipoprotein; HDL, high density lipoprotein; IL28B: interleukin 28B; PNPLA3: patatin-like phospholipase-3; GCKR: glucokinase regulatory protein; LYPLAL1: lysophospholipase-like 1; NCAN: neurocan. Data are given as mean ± standard deviation, median {interquartile range}, or as number of cases (%).

* Histological data are available for all Sicilian patients, and for 126 Northern Italy patients.

Comparing Sicilian patients with the Northern Italian cases with liver biopsy, we have found a more severe spectrum of liver disease, i.e. more severe steatosis, lobular inflammation, fibrosis and obviously a higher prevalence of NASH, but a lower prevalence of hyperferritinemia.

The genetic frequency of the polymorphisms tested in Sicilian and Northern Italian cohorts are reported in Table 1. Specifically, PNPLA3 rs738409 GG genotype was present in 34 (21%) of Sicilian, compared to 33 (13%) of Northern Italy patients (p = 0.02). Genetic frequencies of the five polymorphisms tested in the Sicilian cohort fit with the Hardy–Weinberg equilibrium (χ^2^ = 1.08 for IL28B rs12979860, χ^2^ = 0.10 for PNPLA3 rs738409; χ^2^ = 0.35 for GCKR rs780094; χ^2^ = 0.71 for LYPLAL1 rs12137855; χ^2^ = 0.22 for NCAN rs2228603; p>0.05 for all), as well as genetic frequency of PNPLA3 SNP tested in the Northern Italy cohort (χ^2^ = 0, p>0.05).

### Factors Associated with Carotid Atherosclerosis

The prevalence of carotid plaques was similar in Sicilian and Northern Italian patients (36% vs. 31%; p = 0.29), as well as that of carotid thickening (35% vs. 34% p = 0.83), and mean maximum IMT (0.85±0.24 mm vs. 0.86±0.23 mm; p = 0.66). No study participants had clinically relevant carotid stenosis (i.e., ≥60%).

**Figure 1:**
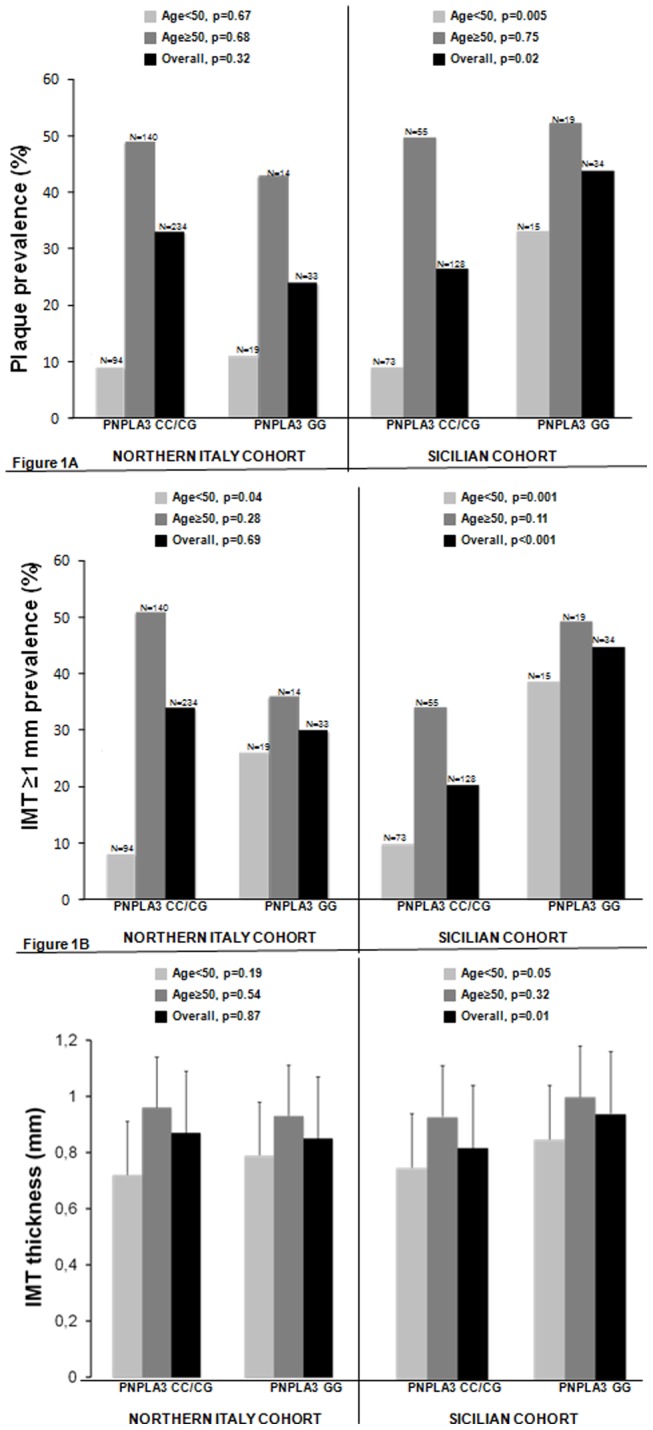
Carotid atherosclerosis in Sicilian and Northern Italian NAFLD patients according to age and to PNPLA3 genotype. Prevalence of carotid artery plaques according to age and to PNPLA3 genotype (A). Prevalence of carotid thickening according to age and to PNPLA3 genotype (B). Mean maximum intima-media thickness values according to age and to PNPLA3 genotype (C).

In the Sicilian cohort the prevalence of carotid plaques and carotid thickening was higher in patients with PNPLA3 GG compared to those with CC and CG genotype (523% *vs.* 32%, p = 0.02; and 62% *vs.* 28%, p<0.001, respectively) (Figure 1A and 1B). The association was only observed in patients younger than 50 (40% *vs.* 11%, p = 0.005 for carotid plaques; and 53% *vs.* 14%, p = 0.001 for IMT thickening), but not in those older than50 years (63% *vs.* 60%, p = 0.75 for carotid plaques; and 68% *vs.* 47%, p = 0.11 for IMT thickening) (Figure 1A and 1B). At multivariate logistic regression analyses, PNPLA3 GG genotype remained independently associated with the above quoted features (OR 2.94; 95% C.I. 1.12–7.70, p = 0.02 for carotid plaques, and OR 3.86; 95% C.I. 1.62–9.15, p = 0.002 for carotid thickening), together with age≥50 years (OR 5.39; 95%C.I. 2.14–13.5, p<0.001) and diabetes (OR 5.29; 95% C.I. 1.51–18.5, p = 0.009) for carotid plaques (Table 2), and with age ≥50 years (OR 4.25; 95%C.I. 1.88–9.62, p = 0.001) and LDL (OR 1.01; 95%C.I. 1.00–1.02, p = 0.007) for carotid thickening (Table 3). After Bonferroni correction, the link between PNPLA3 GG genotype and carotid alterations, was maintained for carotid thickening (p = 0.01), but not for carotid plaques (p = 0.10). Interestingly, no association was found between carotid alterations and both adiponectin serum levels (6.2±2.1 *vs.* 5.9±2.2, p = 0.41 for absence/presence of carotid plaques; 6.1±2.0 *vs.* 6.0±2.3, p = 0.78 for absence/presence of carotid thickening), serum ferritin (258±184 *vs.* 298±361, p = 0.44 for absence/presence of carotid plaques; 245±200 *vs.* 335±364, ng/ml, p = 0.11 for absence/presence of carotid thickening), and the other studied SNPs (Table S1).

**Table 2:** Multivariate Analysis of Risk Factors Associated with the Presence of Carotid Plaques in 429 Italian Patients with Non-alcoholic Fatty Liver Disease.

Sicilian NAFLD Cohort			Northern Italian NAFLD Cohort		
(n = 162)			(n = 267)		
	Multivariate Analysis			Multivariate Analysis	
Variable	OR (95% CI)	*p* value	Variable	OR (95% CI)	*p* value
**Age ≥50** **years**	5.39 (2.14–13.5)	<0.001	**Age >50 years**	7.91(3.72–18.3)	<0.001
**Femal Gender**	2.10 (0.91–4.87)	0.08	**BMI Kg/m2**	1.00(0.97–1.05)	0.95
**Arterial Hypertension**	0.86 (0.34–2.13)	0.75	**Arterial Hypertension**	2.08(1.11–3.89)	0.02
**Type 2 Diabetes**	5.29 (1.51–18.5)	0.009	**ALT– IU/Ml**	1.00(0.99–1.01)	0.86
**Alanine Aminotransferase – IU/L**	0.99 (0.98–1.00)	0.08	**LDL mg/dl**	0.99(0.99–1.01)	0.88
**LDL Cholesterol – mg/dL**	1.00 (0.99–1.01)	0.17	**Ferritin log ng/ml**	1.96(1.41–2.83)	<0.001
**Blood Glucose – mg/dL**	0.99 (0.97–1.00)	0.39	**PNPLA3 GG genotype**	1.15(0.42–3.34)	0.49
**PNPLA3 GG genotype**	2.94 (1.12–7.70)	0.02			
**Lobular inflammation grade 2–3**	1.43 (0.59–3.49)	0.42			
**Severe Liver Fibrosis**	1.09 (0.41–2.91)	0.85			

Abbreviation: IU, international units; BMI, body mass index; LDL, low density lipoprotein; ALT, alanine aminotransferase; PNPLA3: patatin-like phospholipase-3.

**Table 3:** Multivariate Analysis of Risk Factors Associated with the Presence of Carotid Thickening in 429 Italian Patients with Non-alcoholic Fatty Liver Disease, considered overall or according to age.

Sicilian NAFLD Cohort			Northern Italian NAFLD Cohort		
(n = 162)			(n = 267)		
	Multivariate Analysis			Multivariate Analysis	
Variable	OR (95% CI)	*p* value	Variable	OR (95% CI)	*p* value
**Age ≥50** **years**	4.25 (1.88–9.62)	0.001	**Age >50 years**	6.43(3.23–13.6)	<0.001
**Arterial Hypertension**	0.98 (0.41–2.31)	0.96	**BMI Kg/m2**	0.99(0.94–1.03)	0.58
**LDL Cholesterol – mg/dL**	1.01 (1.00–1.02)	0.007	**Arterial Hypertension**	1.39(0.76–2.52)	0.29
**PNPLA3 GG genotype**	3.86 (1.62–9.15)	0.002	**ALT IU/Ml**	0.99(0.98–1.02)	0.49
			**LDL mg/dl**	1.01(1.00–1.02)	0.04
			**Ferritin log ng/ml**	1.19(0.90–1.59)	0.18
			**PNPLA3 GG genotype**	1.32(0.52–3.32)	0.49
			Interaction Age>50*PNPLA3G/G		0.02
**Sicilian NAFLD Cohort <50 years**			**Northern Italian NAFLD Cohort <50 years**		
**(n = 88)**			**(n = 113)**		
	**Multivariate Analysis**		****	**Multivariate Analysis**	
**Variable**	**OR (95% CI)**	***p*** ** value**	**Variable**	**OR (95% CI)**	***p*** ** value**
**Blood Glucose – mg/dL**	1.00 (0.98–1.03)	0.41	**BMI Kg/m2**	0.99 (0.93–1.07)	0.94
**LDL Cholesterol – mg/dL**	1.02 (1.00–1.04)	0.01	**Arterial Hypertension**	1.18 (0.24–7.74)	0.84
**PNPLA3 GG genotype**	7.46 (1.96–28.3)	0.003	**ALT IU/Ml**	0.99 (0.96–1.04)	0.72
			**LDL mg/dl**	1.02 (1.00–1.04)	0.01
			**Ferritin log ng/ml**	1.70 (0.90–2.06)	0.12
			**PNPLA3 148 M/M**	6.00 (1.36–29)	0.01
**Sicilian NAFLD Cohort ≥50 years**			**Northern Italian NAFLD Cohort ≥50 years**		
**(n = 74)**			**(n = 154)**		
**HOMA**	0.89 (0.76–1.05)	0.18	**BMI Kg/m2**	0.99 (0.94–1.03)	0.52
**PNPLA3 GG genotype**	2.13 (0.66–6.83)	0.20	**Arterial Hypertension**	1.46 (0.75–1.81)	0.26
**Grade 2–3 Lobular Inflammation**	0.46 (0.16–1.27)	0.15	**ALT IU/Ml**	0.99 (0.99–1.01)	0.79
			**LDL mg/dl**	1.01 (0.99–1.01)	0.37
			**Ferritin log ng/ml**	1.12 (0.87–1.57)	0.47
			**PNPLA3 148 M/M**	0.54 (0.15–1.78)	0.35

Abbreviation: IU, international units; BMI, body mass index; LDL, low density lipoprotein; ALT, alanine aminotransferase; PNPLA3: patatin-like phospholipase-3.

Among histological features, both moderate-severe lobular inflammation and severe fibrosis were associated with the presence of carotid plaques even if this link was not maintained at multivariate analysis (Table 2). When considering separately patients <50 and ≥50 years, PNPLA3 GG genotype remained independently linked to both carotid plaques (OR 5.00, 95%C.I. 1.24–20.1; p = 0.02) (Table S2) and carotid thickening (OR 7.46, 95%C.I. 1.96–28.3; p = 0.003) (Table 3) in patients younger 50 years only.

In the validation cohort from Northern Italy, the prevalence of carotid plaques and carotid thickening was not significantly different according to the presence/absence of PNPLA3 GG genotype (24% vs. 33%, p = 0.32; and 30% *vs.* 34%, p = 0.69, respectively), but a significant difference was observed for carotid thickening in patients younger than 50 years (26% *vs.* 8%, p = 0.04) (Figure 1A and 1B). When the PNPLA3 GG genotype was forced into the carotid plaque model, the polymorphism was not associated with carotid plaques, while an independent association was observed for age ≥50 years (OR 7.91, 95%C.I. 3.72–18.3, p<0.001), ferritin (OR 1.96, 95%C.I. 1.41–2.83, p<0.001), and arterial hypertension (OR 2.08, 95%C.I. 1.11–3.89, p = 0.02) (Table 2). By contrast, when the PNPLA3 GG genotype and its term of interaction with age was forced into the carotid thickening model, the interaction term (p = 0.02) resulted independently associated with carotid thickening together with age≥50 years (OR 6.43, 95% C.I. 3.23–13.6, p = <0.001) and LDL (OR 1.01, 95% C.I. 1.00–1.02, p = 0.04) (Table 3). Accordingly, considering separately patients <50 and ≥50 years, PNPLA3 GG genotype remained independently associated with carotid thickening in patients younger than 50 years (OR 6.00, 95% C.I. 1.36–29, p = 0.01), but not in their older counterpart (OR 0.54, 95% C.I. 0.15–1.78, p = 0.35) (Table 3).

In the analysis using IMT as a continuous variable, Sicilian patients with PNPLA3 CC/CG genotype had a lower mean maximum IMT than their counterpart with PNPLA3 GG genotype (0.82±0.23 *vs.* 0.94±0.25, p = 0.01). Again, this difference is significant in patients younger than 50 years (0.75±0.17 *vs.* 0.85±0.25, p = 0.05), not in those older (0.93±0.25 *vs.* 1.00±0.26, p = 0.32) (Figure 1C). Older age (p = 0.001), higher LDL cholesterol (p = 0.04) and PNPLA3 GG genotype (p = 0.03) were independent factors associated with high IMT at multiple linear regression analysis. The link between PNPLA3 GG genotype and higher IMT was not maintained after Bonferroni correction (p = 0.15). No association was found in this cohort between IMT and adiponectin, ferritin serum levels, the other studied SNP, and histological features (p>0.10). In Northern Italian patients mean maximum IMT values were similar in patients with and without PNPLA3 GG genotype (0.85±0.21 in GG *vs.* 0.87±0.24 in CC/CG p = 0.87), even if a weak trend was observed in patients <50 years (0.79±0.21 in GG *vs.* 0.72±0.19 in CC/CG, p = 0.19) (Figure 1C). When PNPLA3 genotype was forced in the model, it was not significantly associated with higher IMT (p = 0.32), but IMT was independently linked to older age (p<0.001) and higher LDL serum levels (p<0.001).

**Figure 2:**
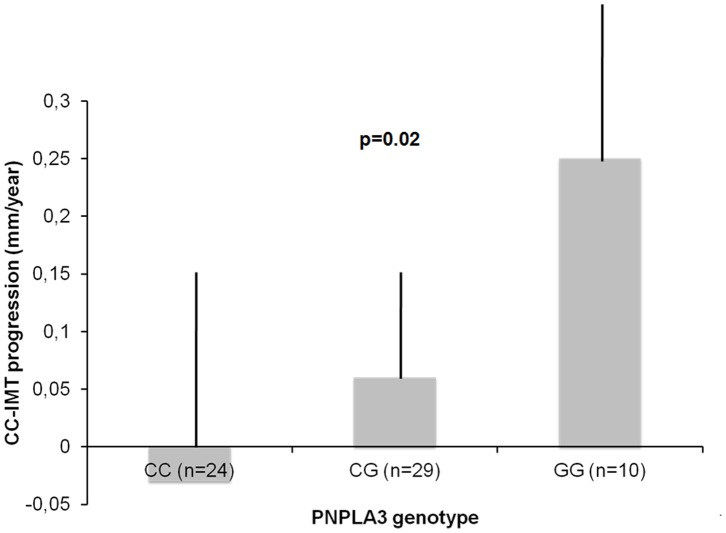
Effect of PNPLA3 CG polymorphism on carotid intima-media thickness progression in 63 Northern Italy patients with NAFLD.

A subgroup of 63 Northern Italy patients underwent ultrasonographic follow-up of carotid assessment. At the baseline, they were characterized by a slightly younger age (age >50, 19/63 = 46% vs. 125/204 = 61% of the rest of the cohort; p = 0.04), and by a lower prevalence of altered iron metabolism (hyperferritinemia, 17/63 = 27% vs. 104/204 = 51%; p<0.001), but with similar sex distribution, BMI, lipid levels, smoking status, liver enzymes and prevalence of NASH, basal IMT thickness and prevalence of plaques (p>0.5 for all). In this cohort, PNPLA3 GG was at risk for IMT progression (Figure 2) compared to CG and CC genotypes (p = 0.02). In particular PNPLA3 GG genotype (p = 0.05), together with age>50 years (p = 0.03) was independently associated with IMT progression by multivariate linear regression analysis (Table 4).

**Table 4:** Independent predictors of CC-IMT progression over time (mm/year) in 63 patients with NAFLD from Northern Italy.

Variable	Estimate coefficient	P value
**Age ≥50 years**	−0.08±0.03	0.030
**Arterial Hypertension**	+0.05±0.03	0.13
**Smoking**	0.06±0.03	0.14
**PNPLA3 GG genotype**	+0.09±0.04	0.050

Abbreviation: PNPLA3: patatin-like phospholipase-3.

## Discussion

In a Sicilian cohort of biopsy-proven NAFLD patients, carotid atherosclerosis was independently associated not only with well-known risk factors for atherosclerosis, but also with the PNPLA3 GG genotype; this association was only observed in younger patients. Of note, this feature was validated in an independent cohort of Northern Italian NAFLD patients, where PNPLA3 GG genotype was also associated with atherosclerosis progression.

Different lines of evidence, including cross sectional and prospective studies, showed that NAFLD patients are at high risk of cardiovascular dysfunction/events, identifying conventional cardiometabolic alterations and the severity of liver damage as risk factors.[2]–[14] To the best of our knowledge this is first study assessing carotid atherosclerosis as a function of gene variants at risk for NAFLD. The novel finding, limited to a cohort of biopsy-proven Sicilian NAFLD patients, is the independent association between the presence of carotid plaques or larger IMT and PNPLA3 GG genotype. Of note this link was maintained after adjustment for well-known cardiovascular risk factors, including lipid levels and IR - not associated in our cohort to PNPLA3 genotype (data not shown) - and histological features of NAFLD.

In particular we found that only 10% of patients <50 years carrying the PNPLA3 CC/CG genotype had carotid plaques, compared with 40% in the group carrying the PNPLA3 GG genotype. This figure is close to the value observed in the subgroup of patients ≥50 years (again, about 60%), irrespective of the PNPLA3 genotype. These data were also confirmed when considering IMT≥1 mm, and high maximum IMT instead of carotid plaques. Interestingly, in the validation cohort of Northern Italian NAFLD patients, vascular alterations were also not different according to PNPLA3 genotype in the total population, but in patients younger than 50 a link between atherosclerosis and PNPLA3 GG genotype was again observed, and maintained after correction for well known risk factors. The weaker association between PNPLA3 genotype and carotid atherosclerosis in Northern Italian, compared to Sicilian patients, could be expression of the lower prevalence of PNPLA3 GG genotype, metabolic dysfunctions and severity of liver disease observed in the validation cohort. In any case, results from both populations are finally consonant, and data from the Northern Italy NAFLD also demonstrated an independent association between PNPLA3 GG genotype and IMT progression over time in a subgroup of patients characterized by young age at presentation.

Although this study was merely observational and not designed to explore the reasons for the association of atherosclerosis markers with PNPLA3 genotype, some hypotheses can be proposed. A recent study by Valenti and colleagues showed that PNPLA3 variant was directly related to hepatic apoptosis in NAFLD. [15] According to these data, it is plausible to hypothesize that PNPLA3 genotype, by regulating apoptotic activity - a finding also involved in the pathogenesis of atherosclerosis [27] -, might modulate vascular damage. PNPLA3 gene variants in individual patients might increase lipid storage in the arterial vessels, similar to that observed in the liver, and could also induce release of ICAM-1, an endothelium-derived inflammatory marker that has been associated with myocardial infarction and stroke. [28] Finally, considering that the mechanisms linking the severity of NAFLD to the PNPLA3 genotype are largely unknown, we might also hypothesize that the same pathway generating liver disease might also produce vascular damage.

The association of PNPLA3 GG genotype with atherosclerosis limited to younger patients is intriguing. A possible hypothesis would be that in younger patients, where the role of aging and classic atherosclerosis risk factors are weaker, PNPLA3 can fully exert its atherogenic role; in older patients, as effect of PNPLA3-induced liver disease progression, the reduction of LDL levels (115.1±38.6 mg/dl in F3–F4 vs. 128.9±38.2 mg/dl in F0–F2 Sicilian cohort) [29] and arterial pressure values associated with advanced liver disease might counterbalance the potential atherogenic role of the PNPLA3 genotype, vanishing the differences in IMT of the different polymorphisms.

We confirmed that type 2 diabetes and higher LDL cholesterol levels in the Sicilian cohort, as well as arterial hypertension, higher LDL cholesterol and ferritin levels in the Northern Italian cohort, were all independent risk factors for carotid atherosclerosis. Interestingly, in the Sicilian population, even if not maintained at multivariate analysis, we also observed an association between carotid plaques and the severity of both liver lobular inflammation and fibrosis, as also reported by Targher et al. [11] Due to the very high prevalence of NASH in the Sicilian cohort, we cannot evaluate the potential association between atherosclerosis and the diagnosis of NASH. Finally, in the test cohort, we did not find any association between carotid atherosclerosis and other SNP promoting NAFLD and its severity. Accordingly GCKR, LYPLAL, NCAN and IL28B gene variants might exert their pathogenic effect mainly in the liver, without (in)directly affecting vascular endothelium.

From a clinical point of view, our data confirm that NAFLD patients are at high risk of carotid atherosclerosis, especially in the presence of classical risk factors, in younger patients with PNPLA3 GG genotype, and in patients aged 50 or more. The lack of association between PNPLA3 GG genotype and atherosclerosis in older patients, together with the selection of a population at high cardiovascular risk (i.e. NAFLD/NASH), could explain why the I148M PNPLA3 gene variant was not identified as a risk factor for cardiovascular diseases/events in GWAS studies, [30], [31] other than a recent report observing a link between ICAM-1 serum levels and PNPLA3 genotype in a large cohort of healthy women. Accordingly, the clinical significance of our data need to be assessed with caution. Further prospective large scale studies, assessing separately younger and older patients are needed to clarify the effect on cardiovascular outcomes, and the importance of a more intensive diagnostic workload and follow-up to prevent cardiovascular complications in selected NAFLD groups according to PNPLA3 genotype.

The main limitation of this study lies in its cross-sectional nature, unable to identify pathogenic mechanisms(s) linking PNPLA3 genotype and carotid atherosclerosis, and in the relative low number of patients younger 50 years. A further methodological question is the potentially limited external validity of the results caused by the small sample of patients especially when considering subgroup analyses according to age. Our study included two cohorts of Italian NAFLD patients, largely overweight-obese, who may be different, in terms of both metabolic features and severity of liver disease, from the majority of prevalent cases of NAFLD in the general population. The lack of data on a control Sicilian population might further limit the strength of our results; however considering the data published from our group, [32] we can observe that our NAFLD population had similar mean IMT and a higher prevalence of carotid plaques, compared to an older control population, [32] and a higher prevalence of PNLA3 GG genotype compared to a cohort of 258 Sicilian CHC patients followed at our center (21%vs 9%) (unpublished data). Obviously, only a case control study could assess if the higher prevalence of carotid atherosclerosis observed in NAFLD [2] compared to individuals without steatosis, is related to an higher prevalence of PNPLA3 genotype and risk of cardiovascular disease. Finally, we also need data on serum levels and liver expression of markers of dysmetabolic function and proinflammatory cytokines potentially involved in the cardiovascular alterations of NAFLD patients.

In conclusion, in two cohorts of Sicilian and Northern Italian NAFLD patients, we showed that PNPLA3 GG genotype is associated with a high risk of carotid atherosclerosis in patients younger than 50. Mechanisms underlying this association, and whether PNPLA3 genotype also influences cardiovascular events need to be further investigated.

## Supporting Information

Table S1 IL28B, GCKR, LYPLAL and NCAN SNPs, and Presence of Carotid Plaques and Carotid Thickening in 162 Sicilian Patients with Non-alcoholic Fatty Liver Disease.
(DOC)

Table S2 Multivariate Analysis of Risk Factors Associated with the Presence of Carotid Plaques in 162 Sicilian Patients with Non-alcoholic Fatty Liver Disease according to age.
(DOC)
