# Supplementary material for: PNPLA3 GG Genotype and Carotid Atherosclerosis in Patients with Non-Alcoholic Fatty Liver Disease
Source: PLoS One. 2013 Sep 17;8(9):e74089. doi: 10.1371/journal.pone.0074089 (PMC3775795; doi:10.1371/journal.pone.0074089)
Supplement: Table S1 — IL28B, GCKR, LYPLAL and NCAN SNPs, and Presence of Carotid Plaques and Carotid Thickening in 162 Sicilian Patients with Non-alcoholic Fatty Liver Disease. (DOC) [file pone.0074089.s001.doc]

**Table S1 1. IL28B, GCKR, LYPLAL and NCAN SNPs, and Presence of Carotid Plaques and Carotid Thickening in 162 Sicilian** Patients with Non-alcoholic Fatty Liver Disease.

| **Variable** | **No Carotid Plaques**  **n=103** | **Carotid Plaques**  **n=59** | **Univariate Analysis**  ***p* value** | **No Carotid Thickening**  **n=105** | **Carotid Thickening**  **n=57** | **Univariate Analysis**  ***p* value** |
| --- | --- | --- | --- | --- | --- | --- |
| **IL28B rs12979860 polymorphism**  TT-TC vs. CC | 55/48 | 33/26 | 0.75 | 55/50 | 33/24 | 0.50 |
| **GCKR rs780094** **polymorphism**  **C**C-CT vs. TT | 73/30 | 35/24 | 0.13 | 72/33 | 35/21 | 0.43 |
| **LYPLAL1 rs12137855** **polymorphism**  **C**C vs. CT-TT | 62/41 | 33/26 | 0.59 | 60/45 | 35/22 | 0.59 |
| **NCAN rs2228603** **polymorphism**  **C**C vs. C/T | 89/14 | 56/3 | 0.10 | 91/14 | 54/3 | 0.10 |

Abbreviation: IL28B: interleukin 28B; PNPLA3: patatin-like phospholipase-3; GCKR: glucokinase regulatory protein; LYPLAL1: lysophospholipase-like 1; NCAN: neurocan. Data are given as number of cases.
