# Supplementary material for: PNPLA3 GG Genotype and Carotid Atherosclerosis in Patients with Non-Alcoholic Fatty Liver Disease
Source: PLoS One. 2013 Sep 17;8(9):e74089. doi: 10.1371/journal.pone.0074089 (PMC3775795; doi:10.1371/journal.pone.0074089)
Supplement: Table S2 — Multivariate Analysis of Risk Factors Associated with the Presence of Carotid Plaques in 162 Sicilian Patients with Non-alcoholic Fatty Liver Disease according to age. (DOC) [file pone.0074089.s002.doc]

**Table S2. Multivariate Analysis of Risk Factors Associated with the Presence of Carotid Plaques in 162 Sicilian Patients with Non-alcoholic Fatty Liver Disease according to age**.

| **Sicilian NAFLD Cohort <50 years**  **(n=88)** | | **Sicilian NAFLD Cohort ≥50 years**  **(n=74)** | |
| --- | --- | --- | --- |
| **Variable** | **Multivariate Analysis**  **OR (95% CI) *p* value** | **Variable** | **Multivariate Analysis**  **OR (95% CI) *p* value** |
| **Female Gender** | 2.72 (0.70 – 10.5) 0.14 | **Type 2 Diabetes** | 3.62 (1.05 – 12.6) 0.04 |
| **Blood Glucose – mg/dL** | 1.02 (0.98 – 1.05) 0.22 | **PNPLA3 GG genotype** | 1.37 (0.45 – 4.19) 0.57 |
| **PNPLA3 GG genotype** | 5.00 (1.24 – 20.1) 0.02 |  |  |

Abbreviation: PNPLA3: patatin-like phospholipase-3.
